# Clinical Characteristics and Genetic Variability of Human Rhinovirus in Mexico

**DOI:** 10.3390/v4020200

**Published:** 2012-01-25

**Authors:** Adriana Landa-Cardeña, Jaime Morales-Romero, Rebeca García-Roman, Ana Georgina Cobián-Güemes, Ernesto Méndez, Cristina Ortiz-Leon, Felipe Pitalúa-Cortés, Silvia Ivonne Mora, Hilda Montero

**Affiliations:** 1 Instituto de Salud Pública, Universidad Veracruzana, Av. Luis Castelazo Ayala s/n, Col. Industrial Ánimas, 91190, Xalapa, Veracruz, México; Email: nita_18lc@hotmail.com (A.L.-C.); jamorales@uv.mx (J.M.-R.); rebgarcia@uv.mx (R.G.-R.); cortiz@uv.mx (C.O.-L.); 2 Instituto de Biotecnología, Universidad Nacional Autónoma de México, Av. Universidad 2001, 62210, Cuernavaca, Morelos, México; Email: acobian@ibt.unam.mx (A.G.C.-G.); sivonnemor@gmail.com (S.I.M.); 3 Hospital Civil ‘Luis F. Nachón’, Secretaría de Salud, Pedro Rendón 1, Esq. Nicolás Bravo, 91000, Xalapa, Veracruz, México; Email: pitalua-felipe1@hotmail.com (F.P.-C.)

**Keywords:** HRV, HRV-C, wheezing, genotyping

## Abstract

Human rhinovirus (HRV) is a leading cause of acute respiratory infection (ARI) in young children and infants worldwide and has a high impact on morbidity and mortality in this population. Initially, HRV was classified into two species: HRV-A and HRV-B. Recently, a species called HRV-C and possibly another species, HRV-D, were identified. In Mexico, there is little information about the role of HRV as a cause of ARI, and the presence and importance of species such as HRV-C are not known. The aim of this study was to determine the clinical characteristics and genetic variability of HRV in Mexican children. Genetic characterization was carried out by phylogenetic analysis of the 5′-nontranslated region (5′-NTR) of the HRV genome. The results show that the newly identified HRV-C is circulating in Mexican children more frequently than HRV-B but not as frequently as HRV-A, which was the most frequent species. Most of the cases of the three species of HRV were in children under 2 years of age, and all species were associated with very mild and moderate ARI.

## 1. Introduction

Infants are one of the populations affected most by acute respiratory infection (ARI). The causes and severity of ARIs have been studied in different populations, and human rhinovirus (HRV) was one of the viruses most frequently associated with ARIs and could be the cause of more than 50% of ARI cases in some pediatric populations [1,2]. In most cases, HRV is associated with self-limiting illness with clinical characteristics similar to those of other viral infections such as fever, rhinorrhea, cough, and wheezing. However, HRV infections could cause complications such as otitis media, bronchiolitis, and pneumonia, which could require hospitalization [1,3,4]. The impact of HRV is not only on ARI; there is evidence that moderate to severe infection caused by HRV is a risk factor for wheezing illnesses and the development of asthma in stages posterior to infection [4]. Therefore, this virus is an important pathogen in public health. 

HRV is an enterovirus that belongs to the family *Picornaviridae*. HRV is an enveloped virus and has a single strand of positive-sense RNA genome of approximately 7.2 kb in length. Serology analysis has detected 99 serotypes of HRV, and nucleotide sequencing analysis of the total and partial HRV genome has been used to classify this virus too. On the basis of that analysis, approximately 150 strains have been found and, until recently, were grouped into two species: HRV-A and HRV-B. Recently, HRV-C, a new genotype, was identified, and another possible species, HRV-D, has been proposed [5,6,7,8]. After the identification of HRV-C, many epidemiologic studies were initiated in different regions of the world to determine the importance of the new HRV species as a cause of ARI. Despite little published information, some data show that HRV-C types have been found in cases with a more severe disease [9,10]. However, this information is not clear, because some studies show that HRV-C could be a cause of mild or moderate disease in the infant population [11]. 

The importance of HRV as a causal agent of ARI in Mexican children has been little studied. This study describes the frequency and clinical characteristics of HRV infection in children under 6 years old and, on the basis of 5′-nontranslated region (5′-NTR) sequencing, determines the species circulating in this population.

## 2. Results

### 2.1. HRV Detection

A screening of 124 nasal swab samples was carried out. In all of the samples, the presence of the HRV-RNA genome was examined by reverse transcription-polymerase chain reaction (RT-PCR) by using primer pairs that encompassed the 400-bp fragment of the 5′-NTR in accordance with a previous report [12]. The samples were obtained from children younger than 72 months old (6 years), the average age was 18 ± 15.6 months, and the 75% of them were not more than 25 months old. Of the total samples, 21 were HRV-positive (21 out of 124, 16.9%). However, most of the HRV-positive cases were in the first or second year of life (18 out of 21, 85.7%); the age range was 2 to 48 months. 

### 2.2. Signs and Symptoms

Clinical manifestations of HRV-positive children were compared with those of HRV-negative children, and the analysis is summarized in Table 1. Note that there are missing data; those data were not found in the medical record of the child or the parents did not provide them. In general, statistically significant differences were not found in the symptoms of the HRV-positive and HRV-negative children. Wheezing, a lower respiratory tract symptom, was found in 42.9% of the HRV-positive cases, and the severity of signs and symptoms of children with wheezing and HRV was not different to a statistically significant degree from that of children without HRV. 

**Table 1:** Clinical characteristics of children with human rhinovirus infection compared with those without this virus.

	HRV-positive	HRV-negative	OR (95% CI)	*P* value
	N = 21	N = 103		
Age in months, median (Min-Max)	15.0 (2.0 to 48.0)	15.0 (0.12 to 67.9)	-	0.99
Younger than 2 years old, n (%)	17 (81.0)	67 (65.0)	2.3 (0.7 to 8.7)	0.16
Female, n (%)	12 (57.1)	47 (45.6)	1.59 (0.6 to 4.5)	0.34
History of asthma, n (%)	0 (0.0)	0 (0.0)	---	---
Previous allergic diseases, n (%)	0 (0.0)	9 (8.7)	---	0.36
Clinical symptoms, n/N (%) [missing values]				
Rhinorrhea	20/21 (95.2) [0]	90/99 (90.9) [4]	2.0 (0.2 to 44.5)	0.99
Cough	19/21 (90.5) [0]	89/95 (93.7) [8]	0.64 (0.1 to 5.0)	0.64
Tearing	6/21 (28.6) [0]	42/101 (41.6) [2]	0.56 (0.2 to 1.7)	0.13
Sore eyes	5/19 (26.3) [2]	25/98 (25.5) [5]	1.04 (0.3 to 3.5)	0.99
Fever >37.5 °C	5/21 (23.8) [0]	33/100 (33.0) [3]	0.63 (0.2 to 2.1)	0.41
Diarrhea	5/21 (23.8) [0]	19/102 (18.6) [1]	1.4 (0.4 to 4.7)	0.56
Wheezing	9/21 (42.9) [0]	55/102 (53.9) [1]	0.6 (0.2 to 1.8)	0.36
Dyspnea	11/21 (52.4) [0]	50/102 (49.0) [1]	1.1 (0.4 to 3.2)	0.78

n/N = subjects with the characteristic of interest/data available. Proportions were compared by using the chi-square or Fisher exact test. Medians were compared by using the Mann-Whitney *U* test. CI, confidence interval; HRV, human rhinovirus; Min-Max, minimum and maximum values; OR, odds ratio.

To analyze the ARI severity caused by HRV, we used a classification that groups the severity of infection into four types: very mild, mild, moderate, and severe [13]. On the basis of the symptoms, 42.85% of the HRV-positive cases were associated with very mild ARI, 52.38% with mild ARI, and 4.76% with moderate ARI. Only one HRV-positive child required hospitalization (1 out of 21, 4.76%). 

### 2.3. HRV Genotyping

For the genotyping of the 21 HRV-positive samples, the products of the 5′-NTR and VP4/VP2 regions amplified by RT-PCR, which have been used previously for the genotyping of this virus [12,14], were sequenced. The 400-bp amplified fragment of 5′-NTR was sequenced in all positive samples of HRV. However, only the 549-bp amplified fragment of VP4/VP2 was detected and sequenced in 7 of 21 HRV-positive samples.

**Figure 1:**
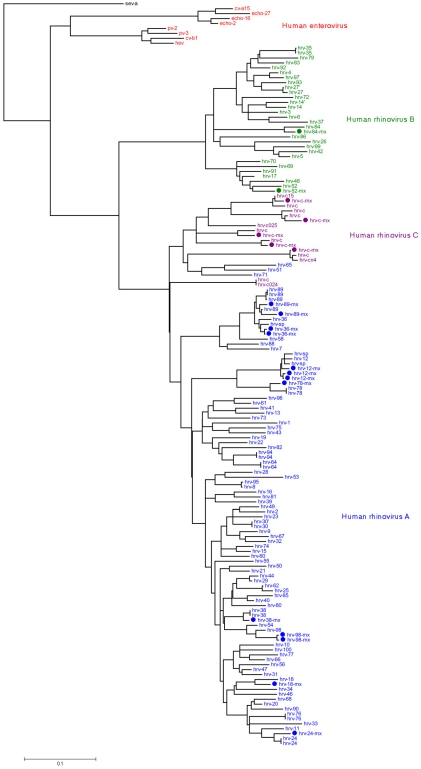
Phylogenetic tree of 5′-nontranslated region (5′-NTR) sequences of human rhinovirus (HRV) from nasal swabs. The phylogenetic tree was constructed by means of the maximum likelihood method. Reference strains of each species obtained from GenBank are indicated, and the species assignment in this study is indicated by circles. Accession numbers are JQ000010 to JQ000029.

The Basic Local Alignment Search Tool (BLAST) was used to conduct a comparative analysis between all of the sequences we obtained and the reported sequences in GenBank. All of the sequences of the 5′-NTR region were used for a phylogenetic analysis (Figure 1). One sample was not included in the construction of a phylogenetic tree, because only 150 nucleotides were identified; however, its sequence was similar to reported sequences, ensuring that the sample corresponded to the HRV-C species. The phylogenetic analysis shown in Figure 1 indicates that the viruses of the three species of HRV—A, B, and C—are circulating in the population of children analyzed. Of the three species of HRV, the A species was the most frequent (61.9%), followed by HRV-C (28.57%) and HRV-B (9.52%). The genotyping of the seven samples obtained by the VP4/VP2 method turned out to be the same as the one obtained by the 5′-NTR method, and so the results were confirmed. 

After the species were found, we analyzed a possible relation between severities of the ARI and the HRV species. The results did not show a statistically significant association between severity and HRV species. However, HRV-A was present in very mild ARI in 35.7% of cases, mild ARI in 57.1% of cases, and moderate ARI in 7.1% of cases. HRV-B was associated only with very mild ARI in 100% of cases, and the newly identified HRV-C was found in very mild and mild ARI in 60% and 40% of cases, respectively. 

## 3. Experiment

### 3.1. Study Design

A cross-sectional study was carried out in children who were under 6 years old and who had ARI during one of two periods. Children in the first period, from November to December 2008, were treated at the ‘Dr. Gastón Melo’ medical unit, and children in the second period, from December 2010 to September 2011, were recruited from the ‘Dr. Luis F. Nachón’ hospital. Both institutions are located in Xalapa, Veracruz, México, which has a latitude of 19°30′43′′N and an altitude of 1,427 m. The study was approved by the ethics committees of the health department of Veracruz state (Secretaría de Salud de Veracruz) and the ‘Dr. Luis F. Nachón’ hospital. The parents of recruited infants in this study provided written informed consent for a sample to be taken and analyzed. The inclusion criteria were age of fewer than 6 years and presentation with an ARI. A structured questionnaire was used to obtain the following information: age, sex, symptoms, asthma and allergy/atopy status, and ARI severity. Severity of illness was divided into four grades—very mild (upper respiratory tract signs or symptoms are present), mild (lower respiratory tract signs or symptoms are present but hospitalization was not required), moderate (lower respiratory tract signs or symptoms are present and hospitalization was required using oxygen saturations in air smaller than 93% on pulse oximetry), and severe (the same characteristics to moderate but the child required oxygen saturations in air more than 93%)—in accordance with the classification by Bezerra and colleagues [13].

### 3.2. Clinical Samples

One hundred twenty-four nasal swabs were collected with sterile rayon-tipped applicators (Puritan Medical Products Co. LLC, Guilford, ME, USA) in accordance with World Health Organization recommendations [15]. Some studies have concluded that there is no significant difference in specificity and sensitivity between nasal swab and nasopharyngeal aspirate specimens when PCR was used for respiratory virus detection [16,17]. The swab was placed into a tube that contained Leibovitz viral transport medium with 100 IU/mL penicillin (American Type Culture Collection, Manassas, VA, USA), 0.01 mg/mL streptomycin, and 0.25 µg/mL amphotericin B (American Type Culture Collection). The samples were maintained at 4 °C and transported to the virology laboratory of the Institute of Public Health at Veracruzana University (Instituto de Salud Pública, Universidad Veracruzana), where the samples were centrifuged at 7,000*g* for 5 minutes and the supernatant was stored at −80 °C until the analysis.

### 3.3. Nucleic Acid Extraction and RT-PCR Assays

Nucleic acids were extracted with a PureLink viral RNA/DNA kit (Invitrogen Corporation, Carlsbad, CA, USA) in accordance with the instructions of the manufacturer. A screening for detecting HRV was carried out by RT-PCR that amplifies the 5′-NTR of HRV genome by means of an AccessQuick™ RT-PCR System (Promega Corporation, Madison, WI, USA) in accordance with the instructions of the manufacturer and with previously described primers and methods [12], and 0.02 µg of HRV-RNA and 0.5 μM of each primer were used. Also, a 549-bp fragment was obtained by amplifying the VP4/VP2 region of the HRV genome in accordance with the previously published primers and protocol [14], except that the alignment was carried out at 42 °C for 1 minute. Finally, amplified PCR products were purified with a High Pure PCR Product Purification kit (Roche, Mannheim, Germany). In all cases, the reference strain HRV 17 (American Type Culture Collection) was used as the positive control.

### 3.4. Nucleotide Sequence Analysis

The purified PCR products were sequenced by the forward primer by means of the Taq FS dye terminator cycle sequencing fluorescence-based sequencing method and a Model 3730 sequencer obtained from PerkinElmer Life and Analytical Sciences, Inc. (Waltham, MA, USA) and Applied Biosystems (Life Technologies, Carlsbad, CA, USA).

### 3.5. Phylogenetic Analysis

The nucleotide sequences were subjected to an online National Center for Biotechnology Information BLAST search, based on this results the species and subtype were defined by a 95% identity or greater (Supplementary Table 1). The best hit was included in the phylogenetic analysis as well as all of the complete rhinovirus genomes available at GenBank. As outgroups, eight human enterovirus sequences were used. This dataset comprises 153 nucleotide sequences. Alignments of 360 nucleotides of the 5′-NTR regions of this dataset were performed with ClustalW, and the evolutionary history was inferred by using the maximum likelihood method based on the Kimura two-parameter model and 1,000 bootstrap replicates. Evolutionary analyses were conducted with MEGA version 5 (Center for Evolutionary Medicine and Informatics, The Biodesign Institute, Tempe, AZ, USA). 

### 3.6. Statistical Analysis

Clinical symptoms, including disease severity, were compared by using the chi-square or Fisher exact test. Age was expressed as a median. Medians were compared by using the Mann-Whitney *U* test. Odds ratio and 95% confidence interval were used to identify the association of these variables with HRV infection. Disease severity was compared for each species. A *P* value not more than 0.05 was considered statistically significant. Statistical analysis was performed with SPSS software version 18 (SPSS Inc., Chicago, IL, USA).

## 4. Discussion and Conclusions

This study shows the frequency of HRV infection in a sample population of children who were under 6 years old and who presented with an ARI. This study is the first report of genetic variability of HRV in a population of children from Mexico (in this case, a part of eastern Mexico with a latitude of 19° 30′ 43′′ N and an altitude of 1,427 m). It is important to mention that, during the collection period, HRV was a cause of ARI principally in children under 2 years old, and signs and symptoms were similar to those of ARIs caused by other pathogens, as was reported in another study [18]. HRV was found in only 17% of the ARI cases in our population. However, different frequencies of HRV as a cause of ARI have been reported [11,19,20]. We consider that the time of year of sampling and the population could be factors affecting the frequency of HRV in our study.

Our study included as many outpatient children as hospitalized children. This allowed us to determine the importance of HRV as a cause of morbidity and to measure the frequency with which HRV could cause symptomatic ARI but not necessarily require hospitalization. In addition, some studies suggest that HRV infection at a young age is a factor associated with the development of asthma or wheezing illnesses, not only when HRV is a cause of moderate or severe ARI [3,4] but also in outpatients who present with wheezing [21,22,23], and the allergic sensitization may play an important role [24]. In the population in this study, HRV was a cause of very mild to moderate infections. It is important to highlight that the severity of signs and symptoms of children with wheezing and HRV was not different to a statistically significant degree from that of children without HRV; this finding is in contrast to that of other studies, in which the clinical characteristics of children with HRV and wheezing were more severe [3]. However, in this study (in accordance with previous studies [21,22]), 42.9% of the children infected with HRV presented with wheezing. These data could suggest that about 45 of every 100 children infected with HRV could develop asthma or wheezing illnesses in posterior stages of infection. This could have a high impact on the quality of life of the infant and their parents and have economic consequences for the family and the health institutions. Given that HRV infection is a cause of morbidity and mortality and that the consequence of HRV infection is a chronic illness, this virus is a great problem in public health. However, the role of HRV as a risk factor to develop chronic illnesses in Mexican children needs to be studied further to establish control strategies to the infection like a rhinovirus vaccine or adequate and opportune treatments. To this end, Lehtinen and colleagues [23] found that the treatment with prednisolone may decrease the probability of recurrent wheezing in a child infected with rhinovirus; this represents a possible alternative for decreasing the problems associated with rhinovirus infection. 

In this study, other viral agents were not detected. However, there are reports that suggest a low probability of co-infections in people with HRV infection and other viral pathogens [25,26,27]. Likewise, there is evidence that HRV and virus co-infections could be associated with a more severe ARI [28,29,30]. In this study, our samples correspond only to very mild to moderate infections, suggesting that HRV single or in co-infection with other pathogens (not detected in this study) did not generate severe ARIs. It is very important that more studies be carried out to address this matter. 

Few reports show the genetic variations of the principal viruses causing ARIs in Mexico. Thus, it is very important to determine the genetic properties of viruses causing ARIs in this population. Our study was limited to an analysis of sequences of specific regions of the HRV genome which have been used for genotyping of the virus, and our interest was to determine the species circulating in the infant population. With this aim, an analysis was carried out with specific regions of 5′-NTR and VP4/VP2 of HRV genome in HRV-positive samples. Of all of the HRV-positive samples examined by 5′-NTR amplification, only 7 were amplified in the VP4/VP2 region. In this study, the method of amplification of the 5′-NTR region was the most efficient, and this is consistent with previous observations [9]. The genotyping obtained by the VP4/VP2 method turned out to be the same as the one obtained by the 5′-NTR method.

The phylogenetic analysis shows that the three species—HRV-A, HRV-B, and the newly identified HRV-C—are circulating in children in the population we analyzed. HRV-C was found with a frequency less than that of HRV-A but greater than that of HRV-B and this also occurred in other populations studied [9]. Interestingly, previous reports suggest that the HRV-C species is more pathogenic since HRV-C infection could cause symptoms of greater severity than HRV-A and HRV-B infections could [9,10,31]. The relation between severity and HRV species associated with the ARI is not clear in this study. Although statistically significant differences were not found, our results, in contrast to those of other works [9,10,31], suggest that HRV-C is associated only with very mild and mild illnesses. 

It is important to mention that the only case of an HRV-positive child who required hospitalization was an HRV-A species. Also, this child had a premature birth, a characteristic described as a possible risk factor of a more severe ARI [32], and this has happened with other respiratory viruses [33]. This suggests that the population characteristics such as the species of the virus causing the infection, plus other possible factors, could have an effect on the severity of the infection.

In conclusion, this study shows that the newly identified HRV-C species is present in the Mexican infant population. The data in this work establish a basis for carrying out new studies in a bigger sample of cases with longer periods of inclusion for a better definition of the molecular epidemiology of this important HRV, and all of its species, in Mexican children. 

## Supplementary Files

Supplementary File 1: PDF-Document (PDF, 68 KB)
