# Supplementary material for: Clinical Characteristics and Genetic Variability of Human Rhinovirus in Mexico
Source: Viruses. 2012 Jan 25;4(2):200–10. doi: 10.3390/v4020200 (PMC3315212; doi:10.3390/v4020200)
Supplement: Supplementary File 1: — PDF-Document (PDF, 68 KB) [file viruses-04-00200-s001.pdf]

Supplementary Table 1. Identity percentages for the NCBI BLAST search of each sequence.

Species	Sequence name	% identity	Name in phylogeny
A	Human rhinovirus 98 5'UTR isolate MX-2011-2A	95	hrv-98-mx
A	Human rhinovirus 18 5'UTR isolate MX-2011-3A	99	hrv-18-mx
A	Human rhinovirus 36 5'UTR isolate MX-2011-4A	98	hrv-36-mx
A	Human rhinovirus 89 5'UTR isolate MX-2011-5A	98	hrv-89-mx
A	Human rhinovirus 78 5'UTR isolate MX-2011-6A	95	hrv-78-mx
A	Human rhinovirus 36 5'UTR isolate MX-2011-8A	98	hrv-36-mx
A	Human rhinovirus 24 5'UTR isolate MX-2011-9A	97	hrv-24-mx
A	Human rhinovirus 12 5'UTR isolate MX-2008-15A	98	hrv-12-mx
A	Human rhinovirus 38 5'UTR isolate MX-2008-18A	98	hrv-38-mx
A	Human rhinovirus 12 5'UTR isolate MX-2008-19A	97	hrv-12-mx
A	Human rhinovirus 12 5'UTR isolate MX-2008-20A	98	hrv-12-mx
A	Human rhinovirus 98 5'UTR isolate MX-2011-21A	96	hrv-98-mx
A	Human rhinovirus 89 5'UTR isolate MX-2011-22A	96	hrv-89-mx
B	Human rhinovirus 84 5'UTR isolate MX-2008-16A	99	hrv-84-mx
B	Human rhinovirus 52 5'UTR isolate MX-2008-17A	99	hrv-52-mx
C	Human rhinovirus C 5'UTR isolate MX-2011-1A	98	hrv-c-mx
C	Human rhinovirus C 5'UTR isolate MX-2011-7A	99	hrv-c-mx
C	Human rhinovirus C 5'UTR isolate MX-2008-11A	97	hrv-c-mx
C	Human rhinovirus C 5'UTR isolate MX-2008-12A	99	hrv-c-mx
C	Human rhinovirus C 5'UTR isolate MX-2008-13A	99	hrv-c-mx
